# Effectiveness of community-based rehabilitation interventions incorporating outdoor mobility on ambulatory ability and falls-related self-efficacy after hip fracture: a systematic review and meta-analysis

**DOI:** 10.1007/s11657-021-00963-0

**Published:** 2021-06-19

**Authors:** Katie J. Sheehan, Laura Fitzgerald, Kate Lambe, Finbarr C. Martin, Sallie E. Lamb, Catherine Sackley

**Affiliations:** 1 Department of Population Health Sciences, School of Population and Environmental Sciences, Kings College London, London, UK; 2 Institute of Health Research, University of Exeter, Devon, UK

**Keywords:** Physiotherapy, Walking, Falls efficacy, Fracture neck of femur, Home-based

## Abstract

***Summary*:**

There is limited evidence from 11 randomised controlled trials on the effect of rehabilitation interventions which incorporate outdoor mobility on ambulatory ability and/or self-efficacy after hip fracture. Outdoor mobility should be central (not peripheral) to future intervention studies targeting improvements in ambulatory ability.

**Purpose:**

Determine the extent to which outdoor mobility is incorporated into rehabilitation interventions after hip fracture. Synthesise the evidence for the effectiveness of these interventions on ambulatory ability and falls-related self-efficacy.

**Methods:**

Systematic search of MEDLINE, Embase, PsychInfo, CINAHL, PEDro and OpenGrey for published and unpublished randomised controlled trials (RCTs) of community-based rehabilitation interventions incorporating outdoor mobility after hip fracture from database inception to January 2021. Exclusion of protocols, pilot/feasibility studies, secondary analyses of RCTs, nonrandomised and non-English language studies. Duplicate screening for eligibility, risk of bias, and data extraction sample. Random effects meta-analysis. Statistical heterogeneity with inconsistency-value (I^2^).

**Results:**

RCTs (n = 11) provided limited detail on target or achieved outdoor mobility intervention components. There was conflicting evidence from 2 RCTs for the effect on outdoor walking ability at 1–3 months (risk difference 0.19; 95% confidence intervals (CI): 0.21, 0.58; I^2^ = 92%), no effect on walking endurance at intervention end (standardised mean difference 0.05; 95% CI: − 0.26, 0.35; I^2^ = 36%); and suggestive (CI crosses null) of a small effect on self-efficacy at 1–3 months (standardised mean difference 0.25; 95% CI: − 0.29, 0.78; I^2^ = 87%) compared with routine care/sham intervention.

**Conclusion:**

It was not possible to attribute any benefit observed to an outdoor mobility intervention component due to poor reporting of target or achieved outdoor mobility and/or quality of the underlying evidence. Given the low proportion of patients recovering outdoor mobility after hip fracture, future research on interventions with outdoor mobility as a central component is warranted.

**Trial registration:**

PROSPERO registration: CRD42021236541

**Supplementary Information:**

The online version contains supplementary material available at 10.1007/s11657-021-00963-0.

## Introduction

Each year, United Kingdom (UK) hospitals admit 70,000 older adults with hip fracture [1]. Even with surgery, there is a fivefold to eightfold increased risk for all-cause mortality in the first 3 months after hip fracture [2]. Among survivors, only 34% regain their pre-fracture mobility (ability to move from and between different postures, e.g. sitting, standing, and walking) by 6-month post-fracture [3]. This may contribute to the reported high rates of transition from independent living to nursing homes among persons with hip fracture [4, 5]. The observed increases in mortality and morbidity led 81 global societies to endorse a call to action for ongoing post-acute care of people whose ability to function is impaired by hip and other major fragility fractures [6].

In a UK qualitative study, patients who were mobile prior to hip fracture identified stability, avoiding falls, and not being afraid of falls during meaningful activities as the outcomes they valued most during their recovery [7]. Indeed, high falls-related self-efficacy and the physical ability to mobilise outdoors are critical outcomes to enable participation in social and family networks and activities [8]. However, up to 65% of older adults report low falls-related self-efficacy after hip fracture [9], and a recent analysis of 24,492 patients indicated a weighted probability of up to 10% for recovery of mobility at 30 days among those able to walk outdoors pre-fracture [10].

To achieve benefits in terms of falls-related self-efficacy and outdoor mobility, a rehabilitation intervention should be tailored to explicitly target improvements in these outcomes [11]. Indeed, a 2010 review reported a potential benefit of psychological intervention on self-efficacy after hip fracture from two RCTs [12]. A previous systematic review identified nine randomised controlled trials (RCTs) of home-based rehabilitation interventions after hip fracture [13]. The authors concluded home-based rehabilitation had considerable positive effect on physical functioning after hip fracture but no effect on walking outdoors [13]. The authors did not describe the extent to which outdoor mobility was incorporated into the home-based rehabilitation interventions identified by their review [13]. Outdoor mobility is likely more physically (gait, strength, and balance), psychologically (confidence, falls-related self-efficacy), and cognitively (navigating environments) challenging than indoor mobility [14]. It is therefore not clear whether the lack of effectiveness was due to an absence of outdoor mobility intervention components across RCTs included in the review [13]. This uncertainty translated to an absence of guidance for interventions to improve falls-related self-efficacy and outdoor mobility after hip fracture in national guidelines [15, 16].

We sought to address this evidence gap by:

1. Determining the extent to which outdoor mobility is incorporated into rehabilitation interventions after hip fracture; and
2. Synthesising the evidence for the effectiveness of these interventions on ambulatory ability (outdoor walking and endurance) and falls-related self-efficacy.

## Methods

### Protocol and registration

This review is reported in adherence to the Preferred Reporting Items for Systematic Review and Meta-analysis statement [17]. The protocol is registered on the International Register of Systematic Reviews (PROSPERO CRD42021236541).

### Eligibility criteria

We included randomised controlled trials (RCT) of community-based rehabilitation interventions which incorporated outdoor mobility for persons after hip fracture. Rehabilitation was defined as ‘a set of interventions designed to optimize functioning and reduce disability in individuals with health conditions in interaction with their environment’ [18]. Rehabilitation interventions for participants after hip fracture are often complex incorporating several interacting components. We employed a broad definition of ‘outdoor mobility’ to determine the extent to which outdoor mobility was captured by these components. This definition included components which targeted going outdoors for structured/unstructured exercise/activity to those which targeted going outdoors for participation, e.g. taking public transport. We included RCTs which planned to incorporate supervised outdoor mobility, unsupervised outdoor mobility, and/or encouragement of outdoor mobility irrespective of whether this was completed by all participants within the RCT. We included RCTs irrespective of comparator group, outcomes measured, length of follow-up, and publication year. We excluded protocols, pilot/feasibility studies, secondary analyses of RCTs, and nonrandomised studies. We excluded RCTs not published in English, due to lack of resources for expert translation.

### Information sources

We searched MEDLINE, Embase and PsychInfo (OVID), CINAHL (EBSCOhost), PEDro, and OpenGrey for published and unpublished RCTs from database inception to 13 January 2021.

### Search

We used a published search strategy based on population, intervention, and study design (hip fracture, rehabilitation, and randomised controlled trials) limited to human and English language (Supplementary File 1) [19].

### Study selection

Three reviewers screened title and abstracts (R1, R2, R3), and two reviewers screened full texts (R1, R3) of potentially eligible RCTs against eligibility criteria. Conflicts were resolved by consensus. Cohen’s Kappa was estimated at k = 0.7 (moderate agreement) for inter-rater reliability prior to consensus work of full-text screening [20].

### Data collection process and data items

Two reviewers (R1, R2) piloted data extraction onto a template adapted from the taxonomy to classify and describe fall-prevention interventions [21]. We sought data for the following data items: author, year, location, sample size intervention group, sample size control group; approach — aim, inclusion criteria, exclusion by dementia/cognitive impairment, other exclusion; base — recruitment, site (s) of delivery, assessment delivered by, intervention delivered by; components — assessment as part of intervention, combination of interventions and description; descriptor intervention — supervised/unsupervised (type, duration, frequency, intensity, individual/group), psychological (cognitive behavioural therapy, other, individual/group), environment, assistive technology, knowledge, post-intervention follow-up (period, type, completeness) and strategies to improve uptake/adherence; descriptor control — routine care/no specific intervention, supervised exercises, medication, knowledge, social environment and other; and outcome — primary, secondary, effect for primary outcome at intervention end and intervention follow-up. Following the pilot, an additional data item specifically related to outdoor mobility was added. One reviewer (R2) extracted the remaining data onto the template. Final extraction was checked for accuracy by a second reviewer (R1). We extracted data from the earliest publication where multiple publications referred to one RCT.

### Risk of bias in individual studies

Two reviewers independently assessed risk of bias at the study level using the Cochrane Risk of Bias Tool (R1, R2) [22]. Conflicts were resolved by consensus.

### Synthesis of results

For our first objective, we reported the extent to which outdoor mobility was incorporated into rehabilitation interventions in a narrative synthesis. For our second objective, we completed an inverse variance random effects meta-analysis to estimate standardised mean difference (for continuous outcomes) or risk difference (for binary outcomes) and their 95% confidence intervals. We interpreted a standardised mean/risk difference of <0.2 as null, 0.2–0.49 as small, 0.5–0.79 as medium and ≥0.8 as large [23]. Statistical heterogeneity was evaluated using the inconsistency-value (I^2^). Results of meta-analysis were presented in tables and forest plots. Meta-analyses were completed in RevMan Version 5.3 (Copenhagen: The Nordic Cochrane Centre, The Cochrane Collaboration, 2011).

### Risk of bias across studies

Small-study publication bias was evaluated through interpretation of funnel plots for each outcome.

## Results

### Selection

**Fig. 1:**
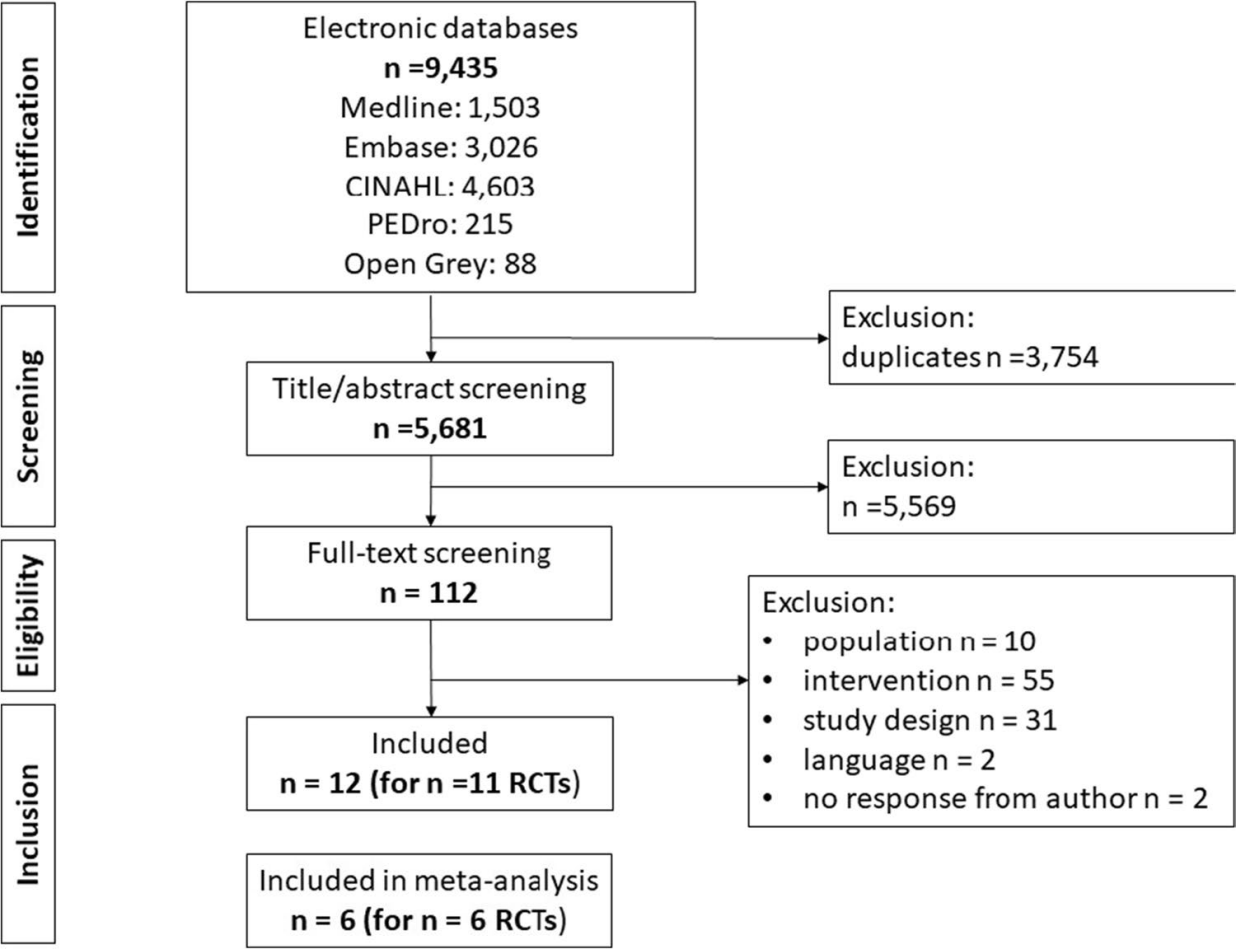
Study selection

We identified 5681 articles after de-duplication. We excluded 5569 on abstract screening. We excluded 99 on full-text screening for nonrandomised study design (n = 31), population (n = 10), intervention (n = 55), language (n = 2), no response from author for additional data related to eligibility (n = 2) and leaving 12 papers reporting 11 RCTs (Fig. 1).

### Risk of bias within studies

**Fig. 2:**
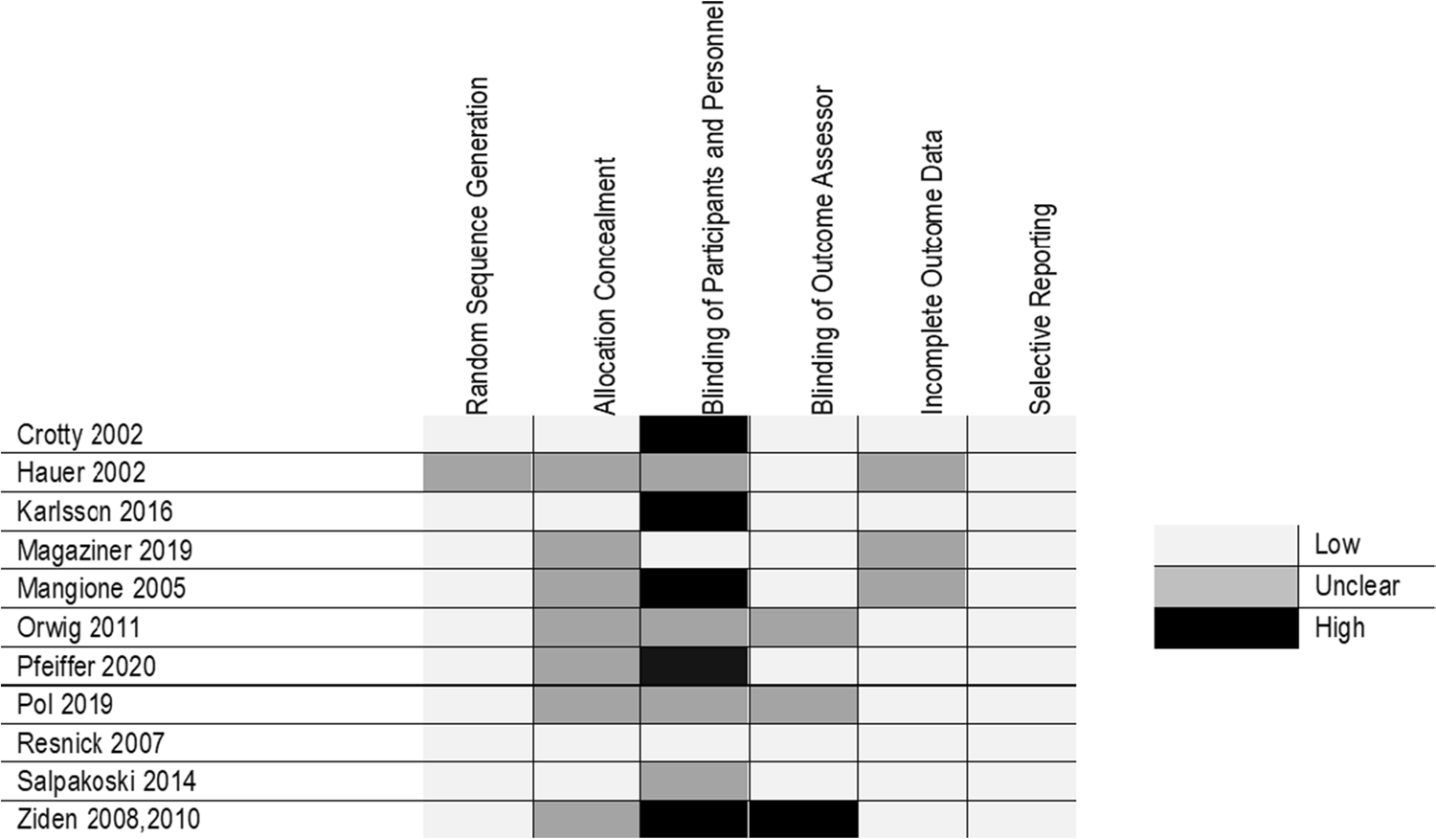
Risk of bias

Most RCTs were at low risk of bias for random sequence generation (n = 10), blinding of outcome assessor (n = 8), incomplete outcome data (n = 8) or selective reporting (n = 11) (Fig. 2). There was insufficient information to assess allocation concealment for 7 RCTs. Lack of blinding of personnel and participants was the most common reason for high bias assignment (n = 5) [24–29]. In addition, one RCT did not blind outcome assessors [24, 25].

### Characteristics of included RCTs

Detailed characteristics for the 11 RCTs are available in Table 1. RCTs were completed in Australia (n = 1) [29], Finland (n = 1) [30], Germany (n = 2) [26, 31], the Netherlands (n = 1) [32], Sweden (n = 2) [24, 25, 28] and the USA (n = 4) [27, 33–35]. Sample size ranged from 28 [31] to 240 participants [32]. Participants were older adults (eligible age range from 60 years plus [26, 30, 33] to 75 years plus [31]) admitted with hip fracture and treated surgically. Nine RCTs excluded potential participants based on their cognitive function [24–27, 29–32, 34, 35]. Karlsson et al. [28] explicitly stated inclusion of participant irrespective of cognitive status, whilst Magaziner et al. excluded participants with ‘low potential to benefit’ or ‘practical impediments to participation’ [33]. Participants were recruited from acute hospital [24, 25, 28, 29, 34, 35], inpatient rehabilitation [26, 31], clinic/health centres [27, 33], nursing and community care facilities [32] or the community [30]. Outcome assessments were completed by physiotherapists [27, 30, 33], occupational therapist [24, 25, 32], gerontologist and psychologist [26], researchers [28] or were not specified [29, 31, 34].

**Table 1:** Characteristics of included RCTs

Author/year	Location	Sample size I:C	Recruitment	Population	Intervention setting	Comparator	Primary outcome	Follow-up
Crotty 2002	Australia	34:32	Acute hospital	Inclusion: ≥ 65 years, medically stable, physical and mental capacity, expected home discharge Exclusion: inadequate social support, no telephone, outside catchment	Home	Routine care	Physical component of Short Form-36	4 months
Hauer 2002	Germany	15:13	Inpatient rehabilitation	Inclusion: ≥ 75 years, female Exclusion: severe cognitive/ cardiovascular/ musculoskeletal disease, acute neurological impairment, unstable chronic/terminal illness, major depression	Outpatient geriatric rehabilitation unit	Seated activities	Muscle strength (1 repetition max, dynamometer, leg press)	3 months
Karlsson 2016	Sweden	107:98	Acute hospital	Inclusion: ≥ 70 years Exclusion: Pathological/ in-hospital fracture, outside catchment	Home	Routine care	Walking independently indoors + outdoors	3 and 12 months
Magaziner 2019	USA	105:105	Clinic/health centres	Inclusion: ≥ 60 years, community dwelling, ambulatory pre-fracture, < 300 m in 6-min walk test at randomisation Exclusion: medically unstable, pathological fracture, low potential to benefit, practical impediments to participation	Home	Seated activities and TENS	300 m or more on 6-min walk test	4 months
Mangione 2005	USA	13:17:11*	Physiotherapy practice	Inclusion: ≥ 65 years, living at home, discharged from physiotherapy, able to travel for assessment Exclusion: MMSE < 20, unstable angina, uncompensated congestive heart failure, metabolic conditions that limit training, residual hemiplegia, Parkinson’s disease, life expectancy of < 6 months, nursing home dwelling	Home	Routine care and written materials	6-min walk test distance	3 months
Orwig 2011	USA	91:89	Acute hospital	Inclusion: ≥ 65 years, female, community dwelling, ambulatory unaided pre-fracture Exclusion: < 20 MMSE, pathological fracture, cardiovascular/neurologic/respiratory diseases/conditions which increase risk of falls limiting exercising home alone, bone disease, metastatic cancer, cirrhosis, end-stage renal disease, hardware in contralateral hip	Home	Routine care	Bone mineral density	2, 6 and 12 months
Pfeiffer 2020	Germany	57:58	Inpatient rehabilitation	Inclusion: ≥ 60 years, community dwelling, positively screened for fear of falling Exclusion: cognitive impairment, severe communication deficiencies	Inpatient rehabilitation and home	Routine care	Short Falls Efficacy Scale and daily walking duration	3 months
Pol 2019	Netherlands	87:76:77†	Nursing and community care facilities	Inclusion: ≥ 65 years, living alone, MMSE ≥ 15 Exclusion: MMSE < 15, terminal illness, awaiting nursing home placement	Home, nursing and community care facilities	Routine care	Canadian Occupational Performance Measure	1, 4 and 6 months
Resnick 2007	USA	51:54:52:51‡	Acute hospital	Inclusion: ≥ 65 years, female, community dwelling, clearance from surgeon Exclusion: MMSE < 20, medical problems that increase falls risk when exercising home alone, walking unaided pre-fracture, pathological fracture	Home	Routine care	Self-efficacy for walking/exercise scale	2, 6 and 12 months
Salpakoski 2014	Finland	40:41	Community — staff of hospital reviewed medical records of admissions	Inclusion: ≥ 60 years, ambulatory pre-fracture, community dwelling Exclusion: MMSE < 8, alcoholism, severe cardiovascular /respiratory disease, progressive disease, severe depression	Home	Routine care and written materials	Ability to negotiate stairs	3, 6 and 12 months
Ziden 2008, 2010	Sweden	48:54	Emergency department	Inclusion: ≥ 65 years, medically approved for geriatric care and rehabilitation, able to speak & understand Swedish Exclusion: documented severe cognitive impairment, severe medical illness with expected survival of < 1 year, severe drug or alcohol abuse, mental illness	Inpatient and home	Routine care and written materials	Falls Self-efficacy Scale (Swedish version)	1 months

*I* intervention, *C* comparator, *MMSE* Mini-Mental State Exam

^*^13 aerobic intervention, 17 resistance intervention, 11 comparator

^†^87 occupational therapy coaching intervention, 76 occupational therapy coaching and sensor intervention and 77 comparator

^‡^51 exercise intervention, 54 motivational intervention, 52 exercise and motivational intervention and 51 comparator

Seven RCTs compared interventions to routine care. This routine care was described as inpatient services, pathways and discharge planning [29]; inpatient rehabilitation for 2–4 weeks [26, 34]; inpatient rehabilitation based on functional needs and a single home therapy evaluation [35]; or interdisciplinary inpatient rehabilitation, discharge planning, referral to ongoing outpatient rehabilitation [24, 25, 28, 32] including handover to physiotherapists/occupational therapists at residential care facilities [28]. Two RCTs provided written materials (home exercise programme [30], non-exercise related written materials [27]) with no further follow-up. Two interventions were compared to sham active controls including seated activities [31], or seated activities and transcutaneous electrical stimulation [33]. Detailed descriptions for each intervention are available in Table 2.

**Table 2:** Intervention descriptors for included RCTs

Author/year	Provider	Supervised/unsupervised	Type	Duration	Frequency	Intensity	Psychological	Environment/assistive technology	Knowledge	Outdoor
Crotty 2002	Multidisciplinary	Supervised	Gait, balance, functional tasks, general physical activity	Individually tailored	Individually tailored	Individually tailored	Goal setting	Home risk assessment, modifications, mobility aids	No	Authorconfirmed outdoor mobility training included
Hauer 2002	Therapeutic recreation specialist	Supervised	Gait, balance, and functional training, strength/resistance, general physical activity	3 months	145 min, 3 days/ week	70–90% max workload	No	No	No	Author confirmed outdoor mobility training included
Karlsson 2016	Multidisciplinary	Supervised	Comprehensive geriatric assessment, gait, balance, and functional training, strength/resistance, general physical activity, monitoring—pain, wound care, medication, nutrition	10 weeks	Initially daily home visits	NA	No	Home risk assessment, modifications, assistive devices	No	Intervention specified walking ability indoors and outdoors
Magaziner 2019	Physiotherapist	Supervised	Gait, balance and functional training, strength/resistance, endurance	4 months	60 min every other day	Strength: 3 × 8 repetitions at 8 repetition max Endurance: 50% heart rate max or 3–5/10 perceived exertion	No	No	No	Intervention specified outdoor ambulation (if able) on flat surface or up and down steps
Mangione 2005	Physiotherapist	Supervised	Group 1: strength/resistance, group 2: endurance	3 months	30–40 min × 2/week month 1 and 2, then × 1/week month 3	Strength: 8 repetition max Endurance: 65–75% heart rate max or 3–5/10 perceived exertion	No	No	No	Intervention specified outdoor and indoor walking included in endurance training
Orwig 2011	Trained non-professionals	Supervised × 3/week, months 1 and 2; × 2/week, months 3 and 4; × 1/1–2 weeks for remainder	Strength/resistance, endurance, flexibility, cognitive behavioural interventions	12 months	Strength × 2/week, 30 min aerobic × 3/week	Strength: 3 × 10 repetitions, × 11 exercises, TheraBand at individual level	Motivational phone calls	No	No	Author confirmed aerobic activity incorporated outdoor walking
Pfeiffer 2020	Physiotherapist, sports therapist	Supervised (8 sessions) and unsupervised	Cognitive behavioural interventions, gait, balance and functional training, strength/resistance	3 months	30–60 min ≥ 2/week	NA	No	Home risk assessment, modifications	Written exercise programme with photos and instructions or recorded instructions with music player, exercise diary	Intervention targeting mobility-based goal example specifies travelling by bus using a wheeled walker
Pol 2019	Occupational therapist	Supervised and unsupervised	Cognitive behavioural interventions, gait, balance and functional training	3 months	60 min/week coaching, on discharge: 4 phone calls over 10 weeks	NA	No	Home risk assessment, modifications	Information and education sessions on importance of physical activity	Specified monitoring of outdoor physical activity; appendix describes case addressing poor outdoor mobility in goal setting
Resnick 2007	Trained non-professionals	Supervised	Strength/resistance, endurance, flexibility	12 months	Strength: × 2/week Aerobic: 30 min × 3/week	NA	Goal setting, group 2 + 3: verbal encouragement, removal of unpleasant sensations, cueing	No	Group 2 + 3 booklet on exercise benefits after hip fracture	Author confirmed aerobic activity incorporated outdoor walking
Salpakoski 2014	Physiotherapist	Supervised (5/6 sessions) and unsupervised	Gait, balance, and functional training, strength/resistance, flexibility, general physical activity	12 months	× 2–3/week	Strength: 3 different strength resistance bands Balance/function: progression	Motivational counselling	Home risk assessment, modifications	Individual non-pharmacological pain management evaluation and interview/discussion of pain-relief strategies, individual motivational face-face + phone call physical activity counselling	Author confirmed functional exercises included outdoor mobility
Ziden 2008, 2010	Multidisciplinary	Supervised and unsupervised	General physical activity, cognitive behavioural interventions, involvement of family in discharge planning	3 weeks	Individually tailored	Individually tailored	Goal setting and motivation	No	No	Physiotherapy intervention focused on improving outdoor mobility

### Synthesis: outdoor mobility in interventions

All 11 RCTs included in this review included outdoor mobility in their intervention. This was explicitly stated by 6 RCTs [24–28, 32, 33] and confirmed with authors for the remaining 5 RCTs [29–31, 34, 35]. Outdoor mobility was supervised [27–29, 31, 33, 35], unsupervised [26, 32] or both supervised and unsupervised [24, 25, 30, 34]. The target duration/distance, frequency, type (e.g. using transport) and/or intensity of outdoor mobility (independent of indoor mobility) was not described for any RCT included in this review. Two authors provided data on the extent to which outdoor mobility was achieved by participants in their RCT [27, 33]. Mangione indicated 83% of participants performed outdoor mobility during the intervention [27]. Cross-sectional data presented at the American Physical Therapy Association, Combined Sections Meeting in 2019 by Mangione et al. [36], indicated the proportion of participants in the larger trial by Magaziner et al. [33] of home exercise after hip fracture who performed outdoor mobility during the home-delivered physical therapy intervention was as follows: visit 3, 44% outdoor walking; visit 8, 57% outdoor walking; visit 16, 62% outdoor walking; visit 24, 63% outdoor walking; and visit 32, 56% outdoor walking (these are for different sample sizes and different seasons). The remaining RCTs did not detail the extent to which outdoor mobility as an intervention component was achieved by participants.

### Synthesis: intervention effectiveness

There was no evidence of publication bias for any of the meta-analyses.

### Ambulatory ability

#### Outdoor walking

**Fig. 3:**
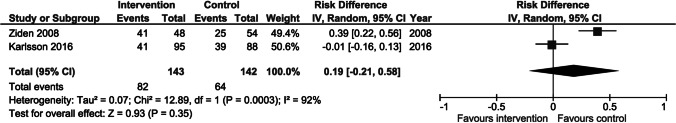
Forest plot illustrating the standardised mean difference and 95% confidence interval of outdoor walking at first follow-up (1–3 months) for rehabilitation interventions with outdoor mobility compared to routine care

Two RCTs selected outdoor walking as their primary outcome. Karlsson et al. defined outdoor walking as the ability to walk independently outdoors [28]. Ziden et al. defined outdoor walking as the ability to walk outdoor alone or with company [24, 25]. These RCTs reported conflicting evidence for the effect of rehabilitation interventions which incorporate outdoor mobility on outdoor walking ability at 1–3-month follow-up (risk difference 0.19; 95% confidence interval (CI): 0.21, 0.58) (Fig. 3) [24, 28]. There was substantial heterogeneity in the analysis I^2^ = 92%. This may be due to systematic differences in participants, interventions and/or target outcomes between the two trials. Karlsson et al. included participants with cognitive impairment and from residential care in their supervised intervention [28]. Ziden et al. excluded potential participants with cognitive impairment and from residential care from their supervised and unsupervised intervention which also incorporated psychological treatment components [24, 25]. Moreover, Ziden et al. explicitly targeted outdoor mobility in their intervention [24, 25].

At 12-month follow-up, there was no between-group difference in the proportion of patients who walked outdoors [25, 28]. Karlsson et al. reported that 48.8% of participants in the intervention group walked outdoors compared to 48.7% of the comparator group, and 90% and 89% participants required a walking device for outdoor ambulation for intervention and comparator group respectively (increase from 69.2% and 65.3% at baseline) [28]. Ziden et al. reported the intervention group recovered outdoor walking by 1-month follow-up, whereas the comparator group recovered outdoor walking by 6-month follow-up [25].

#### Walking endurance

**Fig. 4:**
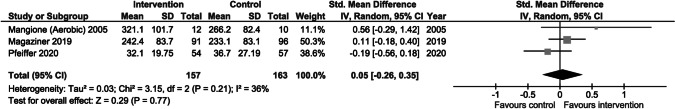
Forest plot illustrating the standardised mean difference and 95% confidence interval of ambulatory ability (6 MWT distance/walking time) at intervention end for rehabilitation interventions with outdoor mobility compared to routine care

Three RCTs selected walking endurance (6-min walk test [27, 33], walking time [26]) as their primary outcome. Rehabilitation interventions which incorporated outdoor mobility were not effective in improving walking endurance at intervention end (standardised mean difference 0.05; 95% CI: − 0.26, 0.35) (Fig. 4). There was low heterogeneity in the analysis I^2^ = 36%.

### Falls-related self-efficacy

**Fig. 5:**
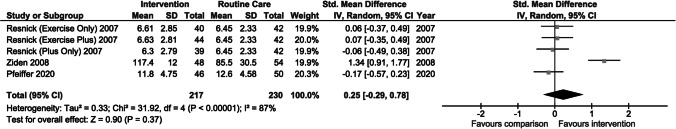
Forest plot illustrating the standardised mean difference and 95% confidence interval of falls-related self-efficacy at 1 − 3 months follow-up for rehabilitation interventions with outdoor mobility compared to routine care

Three RCTs selected falls-related self-efficacy (Falls Self-Efficacy Scale (Swedish version) [24, 25], Short Falls Self-Efficacy Scale [26], self-efficacy for walking [35]) as their primary study outcome. One RCT included falls-related self-efficacy as a secondary study outcome noting a between-group difference at 4-month follow-up favouring the intervention group (median (25th and 75th percentiles): intervention 90.5 (80.5, 98.0) comparison: 79.5 (40.0, 92.5)) [29]. Rehabilitation interventions which incorporated outdoor mobility were suggestive (confidence interval crosses null) of a small increase in falls-related self-efficacy at 1–3-month follow-up compared with routine care (standardised mean difference 0.25; 95% CI: − 0.29, 0.78) (Fig. 5). There was substantial heterogeneity in the analysis I^2^ = 87%. On removal of the RCT by Ziden et al. [24], there was no between-group difference in falls-related self-efficacy at 1–3-month follow-up (standardised mean difference − 0.03; 95% CI: − 0.24, 0.18) and I^2^ = 0%. At 12-month follow-up, there were no between-group differences in falls-related self-efficacy for the study by Resnick et al. [35]. Differences in the Falls Self-Efficacy Scale (Swedish version) observed by Ziden et al. persisted at 6 and 12-month follow-up [25].

## Discussion

### Summary of evidence

We identified 12 papers for 11 RCTs which included outdoor mobility in their rehabilitation intervention for participants after hip fracture. There were methodological concerns related to unblinded participants, personnel, and outcome assessors, and a lack of precision in estimates across included RCTs. Our meta-analyses suggest interventions which include outdoor mobility may be beneficial in terms of outdoor walking and falls-related self-efficacy and not beneficial for walking endurance. However, the RCTs did not provide sufficient detail to replicate the intended outdoor mobility component. Furthermore, most RCTs did not provide detail on the extent to which the outdoor mobility component was actually achieved. Coupled with methodological concerns, we cannot determine the extent to which any potential benefit observed across RCTs may be attributed to an outdoor mobility intervention component.

### Interpretation

The current review identified 7 additional RCTs not included in a previous review of home-based rehabilitation after hip fracture by Wu et al. [13]. We identified the same RCTs by Ziden et al. [24, 25] and Karlsson et al. [28] investigating the effectiveness of interventions on outdoor mobility. Wu et al. proposed no effect on walking outdoors based on these two studies [13]. We adopted a more conservative interpretation of the meta-analysis highlighting the conflicting evidence for effectiveness between the two studies. We also add to the findings of this earlier review by providing results from analysis of both walking endurance and falls-related self-efficacy.

A previous review by Heldmann and colleagues indicated outcome measure selection should be highly specific to the intervention components to reveal benefits attributable to rehabilitation in older patients [11]. For the current review, ambulatory ability and/or falls-related self-efficacy were selected as a primary outcome for 6 of the 11 RCTs identified suggesting outdoor mobility was a peripheral treatment component for half of included RCTs. Indeed, interventions included multiple treatment components many of which do not have a plausible mechanism for changing ambulatory ability or falls-related self-efficacy, e.g. wound care, mediation and nutrition [28]. In addition, most interventions targeted changes in body function/structures through, e.g. resistance training or flexibility (often supervised), as well as changes in activities or participation through (often unsupervised) indoor and outdoor mobility [26, 28, 30, 31, 33, 35]. The peripheral nature of outdoor mobility to these interventions may explain the lack of reported effectiveness on ambulatory ability and falls-related self-efficacy.

Potential benefits in falls-related self-efficacy and/or ambulatory ability were observed for interventions where outdoor mobility was a more central treatment component. The intervention by Ziden et al. focused explicitly on increasing outdoor mobility through physical activity, cognitive behavioural interventions and engagement of family in discharge planning [24, 25]. The alignment between intervention components and outcomes may explain the positive effect (in terms of earlier recovery of outdoor mobility and increased falls-related self-efficacy) observed compared with routine care [24, 25]. The aerobic training arm of the RCT by Mangione et al. was the only intervention to achieve a clinically meaningful (but not statistically significant) between-group difference for the 6-min walk test at the end of the intervention [27, 37]. The observed difference may be attributed to the relevance of the 6-min walk test to an intervention which focused on 20 min of indoor and outdoor walking (83% of participants performed outdoor mobility) at 65 to 75% of age-predicted maximal heart rate [27]. Whilst promising, these interventions were not without methodological concerns. Ziden et al. failed to blind outcome assessors to group allocation which may have led to overestimation of effectiveness [24, 25]. The RCT by Mangione et al. was small with 12 participants in the intervention group and 10 in the control group leading to a lack of precision in outcome estimates. It is therefore not possible to determine whether an intervention with outdoor mobility as a central component leads to benefits in ambulatory ability or falls-related self-efficacy after hip fracture.

Half of RCTs included in this review incorporated a psychological treatment component (goal setting and/or motivation) [24, 25, 29, 30, 34, 35]. Evidence from stroke and primary prevention supports a key role of psychological components in interventions targeting outdoor mobility. For patients post-stroke, a large UK multicentre trial, the ‘Getting Out of The House Study,’ saw a neutral effect of repeated practice of outdoor mobility on outcomes apart from potentially increasing the number of outdoor journeys (secondary study outcome) [38]. The authors noted that the benefit observed was dependent on the treating therapist — indicating a role of motivation and feedback [38]. This is in keeping with an implementation intervention in Australia which reported a beneficial effect of targeting the behaviour of community rehabilitation teams to deliver more outdoor journeys for people post-stroke on the proportion of people achieving outdoor mobility after the intervention [39]. An umbrella review of primary prevention interventions pointed to feedback as a core behaviour change treatment component for increasing physical activity among older adults [40]. For the current review, only one study incorporated objective feedback with the use of sensor output for unsupervised indoor mobility to inform coaching during the intervention [32]. This objective feedback was not extended to outdoor mobility and may be targeted in a future intervention study [32].

There is uncertainty over the external validity of many of the studies included in this review to the underlying population of patients with hip fracture. Most excluded potential participants with cognitive impairment (170 of 1868 (9%) potential participants, where reported) [24, 27, 29, 32, 34, 35], reflecting up to 30% of the underlying population [41]. Only one RCT included participants’ resident in nursing homes [28] where the incidence of hip fracture is high [42]. Moreover, the structure of community-based rehabilitation varies widely regionally, nationally, and internationally. Therefore, it cannot be certain whether results from Australia, Finland, Germany, the Netherlands, Sweden, and the USA may be generalizable to other contexts both within and across countries.

## Strengths and limitations

We used published search terms reviewed by a research librarian. We used broad eligibility criteria with no limitations by characteristics of patients with hip fracture, control group, outcome, length of follow-up or publication date, and used duplicate screening for eligibility and risk of bias, and for a sample set of extracted data to reduce the risk of selection bias. Our broad eligibility criterion for ‘outdoor mobility’ led to identification of intervention components ranging from goal setting related to outdoor mobility to supervised outdoor walking within a target heart rate range. Whilst providing a summary of the existing evidence on outdoor mobility intervention components, this range may have contributed to the statistical heterogeneity observed in meta-analyses. We did not include protocols, pilot/feasibility studies, nonrandomised studies, conference proceedings and/or RCTs not published in English. We excluded two potentially eligible RCTs that we did not receive responses from the authors to determine whether outdoor mobility was included in their rehabilitation intervention [43, 44]. We excluded RCTs not published in English and secondary analyses of RCTs (including 3 secondary analyses of RCTs included in this review [45, 47]). These exclusions may have led to publication bias through the exclusion of evidence relevant to our review question. Finally, we did not assess risk of bias at the outcome level which may have identified additional concerns related to the methodological quality of included studies.

## Conclusions

Previous RCTs incorporated outdoor mobility in their interventions with some indicating a potential benefit in terms of ambulatory ability and/or falls-related self-efficacy after hip fracture. It was not possible to attribute any benefit observed to an outdoor mobility intervention component due to poor reporting of target or achieved outdoor mobility and/or quality of the underlying evidence. Falls-related self-efficacy and the physical ability to mobilise outdoors are critical for patient-reported rehabilitation goals related to participation in social and family networks and activities. Further research on the effectiveness of outdoor mobility interventions after hip fracture on outdoor mobility and known barriers to outdoor mobility (falls-related self-efficacy, falls risk and endurance) is warranted. This research should place outdoor mobility at the centre of an intervention whilst ensuring methodological rigour and addressing challenges for external validity.

## Supplementary Information

Below is the link to the electronic supplementary material.

Supplementary file1 (DOCX 17 KB)

## Data Availability

Not applicable.
