# Supplementary material for: Effectiveness of community-based rehabilitation interventions incorporating outdoor mobility on ambulatory ability and falls-related self-efficacy after hip fracture: a systematic review and meta-analysis
Source: Arch Osteoporos. 2021 Jun 19;16(1):99. doi: 10.1007/s11657-021-00963-0 (PMC8214580; doi:10.1007/s11657-021-00963-0)
Supplement: Supplementary file 1 — Supplementary file1 (DOCX 17 KB) [file 11657_2021_963_MOESM1_ESM.docx]

# Search Strategy

**MEDLINE Search Strategy (Ovid interface)**

1. exp Hip Fractures/

2. exp Femoral Neck Fractures/

3. ((hip* or pertrochant* or intertrochant* or trochanteric or subtrochanteric or extracapsular* or ((femur* or femoral*) adj3 (neck or proximal or head))) adj4 fracture*).mp.

4. 1 or 2 or 3

5. exp REHABILITATION/

6. (rehab$ or (early adj1 (mobil$ or discharg$ or ambulat$)) or occupational therap$ or physiotherap$ or physical therap$ or multidisciplin$).tw.

7. Rehabilitation Nursing/

8. ((geriatric or inter?disciplinary or multi?disciplinary or early or post?operative or post?surgical or home* or intensive or accelerated or intervention or functional) adj2 (intervention or care or rehabilitation or program* or approach or group or recovery)).tw.

9. Rehabilitation/ or Early Ambulation/ or exp Exercise Therapy/ or Occupational Therapy/ or Rehabilitation, Vocational/

10. Health Education/ or Patient Education as Topic/

11. Patient Care/ or Aftercare/ or Ambulatory Care/ or Day Care/ or Postoperative Care/

12. Postoperative Period/

13. Outpatients/

14. Social Adjustment/ or Adaptation, Psychological/

15. Mental Health/

16. Self Efficacy/

17. psychosocial.tw.

18. exp Human Activities/

19. Quality of Life/

20. Social Support/

21. Outcome.mp. and Process Assessment Health care/

22. Health Facilities/ or Ambulatory Care Facilities/ or Community Health Centres/ or Outpatient Clinics, Hospital/ or Rehabilitation Centres.mp.

23. Hospitals, Convalescent/ or Hospitals, Osteopathic/

24. Community Health Services/ or Community Health Nursing/ or Counselling/ or Home Care Services, Hospital-Based/ or Health Services For The Aged/ or Social Work/ or exp Nursing Care/ or Home Care Services/ or Home Nursing/

25. Hospitals, Community/ or Hospitals/

26. exp Comprehensive Health Care/ or Continuity of Patient Care/ or Patient Care Team/

27. (functional status or functional outcome* or ambulation).tw.

28. exp Health Status/ or Recovery of Function/

29. 5 or 6 or 7 or 8 or 9 or 10 or 11 or 12 or 13 or 14 or 15 or 16 or 17 or 18 or 19 or 20 or 21 or 22 or 23 or 24 or 25 or 26 or 27 or 28

30. 4 and 29

31. Randomized Controlled Trial.pt.

32. Controlled Clinical Trial.pt.

33. randomized.ab.

34. placebo.ab.

35. Clinical Trials as Topic/

36. randomly.ab.

37. trial.ti.

38. 31 or 32 or 33 or 34 or 35 or 36 or 37

39. 30 and 38

40. limit 39 to (english language and humans)

**EMBASE and PsychInfo Search Strategy (Ovid interface)**

1. exp Hip Fractures/

2. exp Femoral Neck Fractures/

3. ((hip* or pertrochant* or intertrochant* or trochanteric or subtrochanteric or extracapsular* or ((femur* or femoral*) adj3 (neck or proximal or head))) adj4 fracture*).mp. [mp=title, abstract, original title, name of substance word, subject heading word, keyword heading word, protocol supplementary concept word, rare disease supplementary concept word, unique identifier, synonyms]

4. 1 or 2 or 3

5. exp REHABILITATION/

6. (rehab$ or (early adj1 (mobil$ or discharg$ or ambulat$)) or occupational therap$ or physiotherap$ or physical therap$ or multidisciplin$).tw.

7. Rehabilitation Nursing/

8. ((geriatric or inter?disciplinary or multi?disciplinary or early or post?operative or post?surgical or home* or intensive or accelerated or intervention or functional) adj2 (intervention or care or rehabilitation or program* or approach or group or recovery)).tw.

9. Rehabilitation/ or Early Ambulation/ or exp Exercise Therapy/ or Occupational Therapy/ or Rehabilitation, Vocational/

10. Health Education/ or Patient Education as Topic/

11. Patient Care/ or Aftercare/ or Ambulatory Care/ or Day Care/ or Postoperative Care/

12. Postoperative Period/

13. Outpatients/

14. Social Adjustment/ or Adaptation, Psychological/

15. Mental Health/

16. Self Efficacy/

17. psychosocial.tw.

18. exp Human Activities/

19. Quality of Life/

20. Social Support/

21. Outcome.mp. and Process Assessment Health care/

22. Health Facilities/ or Ambulatory Care Facilities/ or Community Health Centres/ or Outpatient Clinics, Hospital/ or Rehabilitation Centres.mp.

23. Hospitals, Convalescent/ or Hospitals, Osteopathic/

24. Community Health Services/ or Community Health Nursing/ or Counselling/ or Home Care Services, Hospital-Based/ or Health Services For The Aged/ or Social Work/ or exp Nursing Care/ or Home Care Services/ or Home Nursing/

25. Hospitals, Community/ or Hospitals/

26. exp Comprehensive Health Care/ or Continuity of Patient Care/ or Patient Care Team/

27. (functional status or functional outcome* or ambulation).tw.

28. exp Health Status/ or Recovery of Function/

29. 5 or 6 or 7 or 8 or 9 or 10 or 11 or 12 or 13 or 14 or 15 or 16 or 17 or 18 or 19 or 20 or 21 or 22 or 23 or 24 or 25 or 26 or 27 or 28

30. 4 and 29

31. exp Randomized Controlled Trial/

32. exp Controlled Clinical Trial/

33. randomized.ab.

34. placebo.ab.

35. Clinical Trials as Topic/

36. randomly.ab.

37. trial.ti.

38. 31 or 32 or 33 or 34 or 35 or 36 or 37

39. 30 and 38

40. limit 39 to (english language and humans)

**CINAHL Search Strategy (EBSCOhost interface)**

S1. ( (MH “Femoral Fractures+”) or (MH “Femur/SU”) ) or ( femur* or femoral* N3 neck or proximal N4 fracture* ) or ( hip* or pertrochant* or intertrochant* or trochanteric or subtrochanteric or extracapsular* )

S2. geriatric or inter* ormulti* or early or post* or home* or intensive or intervention or functionalN2 intervention or care or rehabilitation or program* or approach or group or recovery

S3. (MH “Rehabilitation+”) or (MH “Geriatric Assessment+”) or (MH “Health Status+”) or (MH “Quality of Life+”)

S4. (MH “Quality of Health Care+”) or (MH “recovery”) or recovery N2 function or ( functional N2 status or outcome* )

S5. S1 and (S2 or S3 or S4)

S6. (MH “Clinical Trials+”) or TI trial or AB ( random* or rct* or placebo )

S7. S5 and S6

**PEDro Search Strategy (available at pedro.org.au)**

1. Abstract & title: fracture*
2. Body part: thigh or hip
3. Method: clinical trial

**OpenGrey Search Strategy**

Fracture
